# Surgical outcomes of robotic bilateral nephrectomy compared to open surgery in adult polycystic kidney disease

**DOI:** 10.1007/s00345-025-05556-x

**Published:** 2025-05-05

**Authors:** Peris Castaneda, John Masterson, Aurash Naser-Tavakolian, Irene Kim, Reiad Najjar, Amit Gupta

**Affiliations:** 1 Department of Urology, Cedars-Sinai Medical Center, Los Angeles, CA USA; 2 Department of Urology, Weill Cornell Medical College, New York, NY USA; 3 Department of Urology, Northwestern University, Chicago, IL USA; 4 Department of Surgery, Cedars-Sinai Medical Center, Los Angeles, CA USA; 5 Department of Medicine, Cedars-Sinai Medical Center, Los Angeles, CA USA; 6 Cedars-Sinai Comprehensive Transplant Center, CA Los Angeles, United States; 7 Beverly Hills Urology, 8631 W 3rd St Ste 531, CA 90048 Los Angeles, United States of America

**Keywords:** Polycystic kidney, Robotic surgery, Nephrectomy, Outcomes research

## Abstract

**Purpose:**

Native nephrectomies for patients with autosomal dominant polycystic kidney disease (ADPKD) have traditionally been performed via an open approach. We have previously described our experience with robotic synchronous bilateral nephrectomies. However, little data is available comparing open nephrectomy (ONx) to robotic nephrectomy (RNx). Here we compare outcomes of ONx and RNx in patients with ADPKD undergoing synchronous bilateral nephrectomy.

**Methods:**

We performed a retrospective review of patients with ADPKD undergoing open or robotic synchronous bilateral nephrectomy from January 2015 to November 2023 at a single institution. Patient characteristics, perioperative factors, kidney size, and complication rates were compared.

**Results:**

Overall, seventeen patients underwent RNx and fifteen patients underwent ONx. There was no significant difference in gender, preoperative BMI, or kidney volume. Patients undergoing ONx had significantly higher estimated blood loss (EBL), length of stay (LOS), and higher rates of non-autologous blood transfusion and complications overall. Complications in the ONx group included 10 patients who required blood transfusions and 2 patients who sustained visceral injuries. Time from nephrectomy to transplant was significantly shorter in the RNx arm.

**Conclusion:**

Robotic synchronous bilateral nephrectomies for ADPKD may have advantages over the traditional open approach, including lower EBL, shorter LOS, decreased overall complication rates, less severe complications and potentially faster time from nephrectomy to transplant.

## Introduction

Autosomal dominant polycystic kidney disease (ADPKD) is the most common hereditary disorder of the kidney, affecting approximately 4 to 7 million people worldwide [1]. ADPKD is characterized by the formation of fluid-filled cysts that encroach on the renal parenchyma and progressively impair renal function. Up to 75% of patients with ADPKD will progress to end-stage renal disease (ESRD) by age 70 [2]. In the United States, ADPKD accounts for 10–15% of patients requiring dialysis [1]. 

Common indications for nephrectomy in patients with end stage ADPKD include creation of space for a renal transplant, hematuria, recurrent infection of cysts or the urinary tract, and symptoms caused by the mass effect of the enlarged kidneys [3]. 

Native nephrectomy in ADPKD has traditionally been performed using an open approach. However, open bilateral nephrectomy is associated with high rates of major complications, including blood loss, prolonged hospitalization and ileus, and hypotension [4]. With the contemporary shift toward minimally invasive surgery, laparoscopic and hand-assisted approaches to native nephrectomy in ADPKD have become increasingly utilized. Studies have shown that laparoscopic synchronous bilateral nephrectomy offers decreased morbidity, shorter hospital stay, and reduced postoperative pain compared to an open approach [5, 6]. 

However, a laparoscopic approach is technically demanding, particularly in cases of massively enlarged kidneys [7, 8]. Recently, robotic-assistance has been applied to surgeries previously performed open or laparoscopically including nephrectomies for malignant conditions. A robotic approach to native nephrectomy in ADPKD may offer the advantages of minimally invasive surgery while improving mobility and technical feasibility in extremely large kidneys. We have previously reported our series of 14 patients undergoing robotic bilateral synchronous nephrectomy, and found improved outcomes even for very large kidneys [9]. 

Although a few studies have compared laparoscopic and open approaches, to our knowledge, there have been no studies directly comparing robotic and open bilateral synchronous nephrectomy in ADPKD. Here we compare patient factors and perioperative outcomes in patients with ADPKD undergoing open (ONx) or robotic (RNx) bilateral nephrectomy.

## Methods

After Institutional Review Board approval was obtained, we performed a retrospective review of all patients undergoing open or robotic bilateral nephrectomy for ADKPD at a tertiary care center between January 2015 and November 2023. Patient demographics were collected along with preoperative data on presenting symptoms and indication for nephrectomy. Pre-operative cross-sectional imaging was reviewed when available, and the dimensions of each kidney were measured. All RNx surgeries were performed by a single surgeon (AG) with extensive robotic experience. Our surgical technique has been previously described [9]. The ONx surgeries were performed by 5 different surgeons of both transplant and urologic specialties, all of whom were fellowship-trained.

Intraoperative factors included operative time and estimated blood loss (EBL). Intraoperative and post-operative complications were reviewed, as well as readmission rates. Data from the two groups were compared and assessed for statistical significance (*p* < 0.05).

## Results

### Patient demographics and kidney size

Within the specified time frame, seventeen patients underwent RNx and fifteen patients underwent ONx (Table 1). The median ages of patients undergoing RNx and ONx were 50.4 (IQR 45.5–57.1) and 57.9 (IQR 54.1–65.3), respectively (*p* = 0.03). The RNx group had a higher proportion of women (64.7% vs. 33.3%) and a higher median BMI (24.6 vs. 23.4) compared to the ONx group, though neither of these was found to be statistically significant (*p* = 0.08 and *p* = 0.22, respectively). The most commonly cited indications for nephrectomy were to create space for transplant (RNx– 58.8%, ONx– 73.3%), the presence of symptoms (RNx– 58.8%, ONx– 66.7%), and recurrent pyelonephritis (RNx– 17.6%, ONx– 6.7%). For two patients in the ONx group (13.3%), concern for renal cell carcinoma was also noted as an indication. Symptoms included flank pain, abdominal pain, early satiety, recurrent urinary tract infections, infection of cyst, hematuria, shortness of breath, and limitations in mobility.

**Table 1:** Pre-operative characteristics and kidney size

	Robotic Nephrectomy (*n* = 17)	Open Nephrectomy (*n* = 15)	*p*-value
**Female** (n, %)	11 (64.7%)	5 (33.3)	0.08
Age (median, IQR)	50.4 (45.5–57.1)	57.9 (54.1–65.3)	0.03*
BMI (median, IQR)	24.6 (22.9–30.0)	23.4 (20.4–28.6)	0.22
**Indication for surgery**			
To create space for transplant	10 (58.8%)	11 (73.3%)	
Symptomatic	12 (58.8%)	10 (66.7%)	
Recurrent pyelonephritis	3 (17.6%)	1 (6.7%)	
Concern for RCC	0 (0%)	2 (13.3%)	
**Largest kidney dimension on preoperative CT (cm)** **(median**,** IQR)**			
Right Kidney (range)	13.9–32.8	20.7–33.9	
Right Kidney (med, IQR)	24.0 (19.2–25.3)	27.7 (22.8–32.3)	0.04*
Left Kidney (range)	13.7–30.0	17.9–35.3	
Left Kidney (med, IQR)	21.2 (18.3–25.2)	24.3 (22.0–29.9)	0.07
**Ellipsoid kidney volume (cm3)**			
Unknown	0 (0%)	4 (26.7%)	
Right Kidney (range)	276–6603	972–5618	
Right Kidney (med, IQR)	2504 (1021–3448)	2014 (1885–3842)	0.43
Left Kidney (range)	302–4754	990–6558	
Left Kidney (med, IQR)	2162 (1421–3267)	2429 (1272–5623)	0.61

Preoperative cross-sectional imaging was available for seventeen (100%) patients who underwent RNx and eleven (73.5%) patients who underwent ONx. Each kidney was measured in its largest dimension craniocaudally; length, width, and height of each kidney were also measured, from which the ellipsoid volume was calculated. Median largest dimension of the right kidney was 24.0 cm in the RNx group and 27.7 cm in the ONx group (*p* = 0.04). Median largest dimension of the left kidney was 21.2 cm in the RNx group and 24.3 cm in the ONx group (*p* = 0.07). Median volume for the right kidney was 2504 cm^3^ in the RNx group and 2,014 cm^3^ in the ONx group (*p* = 0.43). Median volume for the left kidney was 2,162cm^3^ in the RNx group and 2,429 cm^3^ in the RNx group (*p* = 0.61).

### Perioperative

There was no significant difference in operative time between the two groups. Those undergoing RNx had a median operative time of 311 min, compared to 279 min in those undergoing ONx (*p* = 0.13). The ONx group had a higher median EBL (500mL, IQR 175–850) compared to the RNx group (100mL, IQR 50–200; *p* = 0.005). The median length of stay for the ONx group was 8 days (IQR 4–11) compared to 3 days in the RNx group (IQR 2.5–6.5; *p* = 0.02). A higher proportion of people in the ONx group required admission to the ICU post operatively (40.0% compared to 11.8% in the RNx group; *p* = 0.11). Finally, four patients (26.7%) who underwent ONx required a nasogastric tube (NGT), while no patients who underwent RNx required one.

### Post-operative complications and readmissions

Two patients (11.8%) in the RNx group had at least one post operative complication compared to ten patients (66.7%) in the ONx group (*p* = 0.003). (Table 2) Only one patient (5.9%) in the RNx group required intraoperative or post-operative non-autologous blood transfusions, compared to ten patients (66.7%) in the ONx group (*p* < 0.001). No visceral injury occurred in the RNx group, whereas two patients (13.3%) in the ONx group sustained a visceral injury. Of these two patients, one patient sustained a liver laceration and diaphragmatic injury, both of which were primarily repaired. The second patient sustained lacerations to the spleen and diaphragm, which were primarily repaired, and an injury to the common bile duct which required wedge resection of the liver and endoscopic retrograde cholangiopancreatography (ERCP). This patient subsequently developed a pleural effusion due to the diaphragmatic injury, which required thoracoscopic surgery. Only one patient who underwent RNx (5.9%) and one patient who underwent ONx (6.7%) experienced an abdominal wall hematoma or seroma. Surgical site infection (SSI) occurred in one patient (5.9%) who underwent RNx; however, no patients who underwent ONx experienced SSI. There were no instances of incisional hernia found to be related to the procedure in either group. Notably, for two patients in the ONx group, the help of a plastic surgeon was elicited to close the fascial defect.

**Table 2:** Perioperative factors and Post-Operative complications

	Robotic Nephrectomy (*n* = 17)	Open Nephrectomy (*n* = 15)	*p*-value
**Peri-operative details**			
Operative time in minutes, (median, IQR)	311 (276–391.5)	279 (195–379)	0.13
Estimated blood loss (mL), (median, IQR)	100 (50–200)	500 (175–850)	0.005*
Length of stay (days), (median, IQR)	3 (2.5–6.5)	8 (4–11)	0.02*
ICU stay	2 (11.8%)	6 (40.0%)	0.11
ICU days (median)	1.5	1.5	
Required NGT	0 (0%)	4 (26.7%)	
**Complications**			
Any	2 (11.8%)	10 (66.7%)	0.003*
Non-autologous blood transfusion	1 (5.9%)	10 (66.7%)	< 0.001*
Units transfused (median, IQR)	1 (--)	2 (2, 4)	
Abdominal wall hematoma/seroma	1 (5.9%)	1 (6.7%)	
Surgical wound infection	1 (5.9%)	0 (0%)	
Visceral Injury	0 (0%)	2 (13.3%)	
30-day readmission	2 (11.8%)	3 (20.0%)	0.65
Months from nephrectomy to transplant (days), (median, IQR)	2.7 (1.5–6.1)	7.6 (4–16)	0.04*
**Pathology**			
Benign	16 (94.1%)	13 (86.7%)	
Malignant	1 (5.9%)	2 (13.3%)	

Within 30 days of discharge, two patients (11.8%) in the RNx group and three patients (20.0%) in the ONx group were readmitted (*p* = 0.65). In the RNx group, one patient was readmitted for flank bruising and altered mental status due to uremia. The second patient was readmitted for a wound complication. In the ONx group, the patient was readmitted for the development of a pleural effusion resulting from a diaphragmatic injury during surgery; another patient was readmitted for altered mental status; and the third patient was readmitted for shortness of breath, volume overload, and hyperkalemia.

### Renal transplant

Among those who underwent ONx, nine patients (60.0%) had renal transplantation after their nephrectomy. Three patients had undergone a renal transplant prior to their native nephrectomy: one patient had undergone a transplant approximately one year prior and the remaining two patients had a remote history (> 5 years) of renal transplant. The remaining patients have not undergone renal transplant to date. Among those who underwent RNx, nine patients (53.0%) had renal transplantation after their nephrectomy. Two patients had renal transplant prior to their native nephrectomy and six have not undergone renal transplant. The median time from nephrectomy to transplant was 2.7 months (IQR 1.5–6.1) in the RNx group compared to 7.6 months (IQR 4–16) in the ONx group (*p* = 0.04).

## Discussion

Native nephrectomies are performed for patients with end stage ADPKD who experience symptoms or who require room for a renal transplant. While this procedure has traditionally been performed via an open approach, exceptionally high morbidity rates have been reported. Laparoscopic and hand-assisted approaches have gained popularity in recent years but tend to be technically difficult, particularly with larger kidneys. A robotic approach offers the advantages of minimally invasive surgery with improved dexterity and mobility compared to laparoscopy [9]. In this study, we compared patients with ADPKD undergoing synchronous bilateral nephrectomies via an open approach and a robotic approach.

In our study, open nephrectomy was associated with a higher rate of overall complication (67% compared to 12%), the most frequent of which was perioperative non-autologous blood transfusion, which occurred in 67% of patients in the open group and 6% in the robotic group. These findings are consistent with prior series of ONx in ADPKD. An early study by Bennet et al. described a 38% rate of severe complications and a 3% mortality rate [10]. In a series of 33 patients undergoing ONx, Bellini et al. reported a perioperative transfusion rate of 42% [11]. Not only was ONx associated with a higher rate of complications than RNx in our study, but the complications encountered were more severe. Visceral injury did not occur in any patients who underwent RNx. However, multiple visceral injuries occurred in two patients undergoing ONx, and these injuries required secondary procedures to repair.

Comparative studies of open and laparoscopic approaches have shown reduced complications with the latter. Verhoest et al. reported a series of 21 patients who underwent laparoscopic nephrectomy and 19 who underwent open nephrectomy [12]. Those undergoing laparoscopic surgery had a lower rate of post-operative blood transfusion (4.8% compared to 15.8%, significance not reported). Guo et al. performed a systemic review and meta-analysis of patients across seven studies undergoing laparoscopic and open nephrectomy for ADPKD [13]. The authors found significantly fewer perioperative complications in the laparoscopic group (RR 0.545, *p* = 0.018).

In addition to lower rates of overall complication, our study found RNx to be associated with a shorter hospital stay (median time 3 days vs. 8 days in the ONx group; *p* = 0.02) and less time to renal transplant (median time 2.7 months vs. 7.6 months; *p* = 0.04). A difference of over 4 months is likely to be clinically significant and have great impact on quality of life, given the complications and high rate of side-effects associated with prolonged hemodialysis [14, 15]. The LOS in our ONx group is consistent with that of Bellini et al. who reported a median hospital stay of 9 days in their open series [11]. Comparative studies of open and laparoscopic nephrectomy have similarly shown a decreased LOS and time to renal transplant. Verhoest et al. showed an average hospital stay of 8.3 days in their open group compared to 5.2 days in their laparoscopic group (*p* = 0.002) [12]. Notably, the authors also reported a decreased delay between nephrectomy and renal transplant, 12 months in the open group and 3.1 months in the laparoscopic group, though significance level was not reported.

Laparoscopy therefore promises certain advantages over open nephrectomy for ADPKD. However, there are several limitations to the laparoscopic approach. The massive size of kidneys in ADPKD can render laparoscopy technically challenging or, at times, unfeasible. In a series of 18 patients undergoing had-assisted laparoscopic nephrectomy, Lipke et al. saw an open conversion rate of 22%, associated with kidney size [7]. The authors found that conversion to ONx was more likely in kidneys that exceeded 3,500 cm^3^. Ivey et al. reviewed patients undergoing laparoscopic nephrectomy across all indications. In their series, 44 of 399 patients required open conversion. The authors noted that ADPKD was associated with the highest rate of conversion (13%) and found a 40% conversion rate among large kidney specimens weighing over 1,500 g [16]. 

Robotic surgery may provide the dexterity and exposure needed to remove very large kidneys without conversion to open surgery. In our series, median kidney volume among those undergoing RNx was 2,450 cm^3^ (IQR 1,021–3,448) in the right kidney and 2,162 cm^3^ (IQR 1,421–3,267) in the left kidney, with no significant difference in volume between the two groups. In the RNx group, median largest kidney dimension was 24.0 cm (IQR 19.2–25.3) in the right kidney and 21.2 cm (18.3–25.2) in the left kidney. These values are comparable to the dimensions reported in laparoscopic series. A study by Chen et al. compared outcomes of laparoscopic and open bilateral nephrectomy in ADPKD [17]. Median preoperative kidney volume in the laparoscopic group was 1,050 cm^3^ in the right kidney and 837 cm^3^ in the left kidney, without significant difference in size in the open group. Two patients (6%) in the laparoscopic group required conversion to open due to adhesions. In a series of 19 patients undergoing either unilateral or bilateral laparoscopic nephrectomy by Bendavid et al., median kidney size was 22 cm [18]. The authors saw an 18% rate of conversion from laparoscopic to open, due to bleeding, adhesions, or vascular/visceral injury. Despite the large kidney sizes of patients undergoing RNx in our study, no patients in the robotic group required conversion to open nephrectomy.

Furthermore, our series showed no significant difference in median operative time between those undergoing RNx and ONx. Several comparative studies of laparoscopic vs. open nephrectomy in ADPKD have shown significantly increased operative time with a laparoscopic approach. In a series by Gil et al., average surgical time for patients undergoing laparoscopic bilateral nephrectomy was 36 min longer than for those undergoing open nephrectomy (4.4 vs. 3.8 h, *p* = 0.007), despite no significant difference in kidney size between the two groups [5]. Lubennikov et al. found a median operative time of 218 min (IQR 197.5–305) in those undergoing laparoscopic bilateral nephrectomy compared to 115 min (IQR: 107.5–145) in those undergoing open bilateral nephrectomy (*p* < 0.001) [19]. In both cases, authors attribute some of the time difference to repositioning of the patient in order to perform the contralateral nephrectomy. Our open operative times are slightly higher than those reported in the literature; however, our robotic operative times are shorter than those reported in a recently published series, both of which may reflect institutional variations [20]. 

Those who underwent ONx had a higher rate of blood transfusion than those who underwent RNx. Consequently there is concern for increased sensitization in these patients with end stage ADPKD who will ultimately require renal transplant [21]. 

Our study has certain limitations to be acknowledged. First, we describe a retrospective series, with all bilateral RNx performed by a single surgeon. Indeed, a robotic approach to synchronous bilateral nephrectomy in ADPKD is relatively novel, and, to our knowledge, this is the largest robotic bilateral nephrectomy series and the only comparative study reported to date. Perioperative complication rates may be expected to vary depending on surgeon experience and proficiency. Secondly, our two cohorts saw significant differences in age (50.4 in RNx vs. 57.9 in ONx) and largest dimension of the right kidney (24.0 cm in RNx vs. 27.7 cm in ONx); due to the low number of patients, we were not able to adjust for these factors on outcomes analysis. Of note, there was no significant difference in right or left kidney volume between the two groups. Finally, most of our robotic nephrectomies were performed in the past few years, and long-term outcomes are not yet available. However, given that the majority of complications reported in the literature occur in the immediate or short-term post-operative period, and our robotic approach is associated with improved post-operative morbidity, we do not expect unique long-term complications.

## Conclusion

Robotic synchronous bilateral nephrectomy is a comparatively safe and effective approach in patients with end stage ADPKD, with several advantages over open surgery. These include lower rates of blood transfusion, reduced complications, and shorter duration of hospital stay. This technique can be performed in large kidneys without increased operative time. A robotic approach should therefore be considered whenever nephrectomy is indicated for those with ADPKD.

## Data Availability

No datasets were generated or analysed during the current study.
